# Efficacy of anti-VEGF monotherapy versus anti-VEGF therapy with subthreshold micropulse laser (SML) in the management of diabetic macular oedema (DMO): a systematic review and meta-analysis

**DOI:** 10.1007/s00417-024-06405-0

**Published:** 2024-02-29

**Authors:** Chandana Wijeweera, Jing Ni, Peter Petocz, Veronica Preda, James Jabbour

**Affiliations:** 1 Faculty of Medicine, Health and Human Sciences, Macquarie University Macquarie Park, Sydney, Australia; 2 Graduate Research Academy, Macquarie University Macquarie Park, Sydney, Australia; 3 Sydney Eye Specialists, Sydney, Australia

**Keywords:** Diabetic macular oedema, Diabetic retinopathy, Conventional laser, Anti-vascular endothelial growth factor, Subthreshold micropulse laser therapy, Central macular thickness

## Abstract

**Background:**

Intravitreal injection anti-vascular endothelial growth factor (IVI anti-VEGF) therapy serves as the primary treatment for centre involving diabetic macular oedema (DMO). Conventional laser therapy (CLT) adjunct has proven beneficial; however, it is not widely used due to significant risks of retinal scarring. Subthreshold micropulse laser (SML) therapy has, however, emerged as a comparable alternative to combination therapy, offering a distinct advantage by mitigating the risk of retinal scarring.

**Methods:**

A search of six databases was conducted. A meta-analysis of mean differences was performed including subgroup analyses where appropriate. Primary outcome was the number of injections at 12–14 months; secondary outcomes were changes in central macular thickness (CMT) and best corrected visual acuity (BCVA) at 6–8 months and 12–14 months.

**Results:**

A total of ten papers including six randomised clinical trials and four retrospective clinical studies were included in our study, capturing 563 eyes of 478 patients. Overall, the risk of bias was moderate for these studies. Significantly fewer anti-VEGF therapy injections were administered in the combination therapy versus anti-VEGF monotherapy patients at 12–14 months who had poor visual acuity (6/18 Snellen or worse) at baseline, mean difference − 2.25 (95% CI; − 3.35, − 1.15; *p* < 0.05). Combination therapy was not associated with significantly fewer intravitreal injections in patients with a higher visual acuity (6/15 Snellen or better) at baseline. Our analysis also showed significant improvements to both BCVA and CMT were reached at 6 − 8 month post-baseline at the 95% confidence intervals: − 1.13 (− 2.09, − 0.16) and − 4.04 (− 7.59, − 0.50). These improvements remained statistically significant at 12–14 months: − 0.94 (− 1.67, − 0.20) and − 1.92 (− 3.52, − 0.32) respectively with combination therapy.

**Conclusion:**

Our findings demonstrate that combination therapy (SML + IVI anti-VEGF) is associated with fewer intravitreal injections. We report a better BCVA and a reduction in CMT at 6 and 12 months from baseline with combination treatment compared to the IVI anti-VEGF monotherapy comparator. SML is a proven non-scarring cost-effective therapy for DMO that should be readily available in the medical retinal therapy as it may reduce the burden of care.


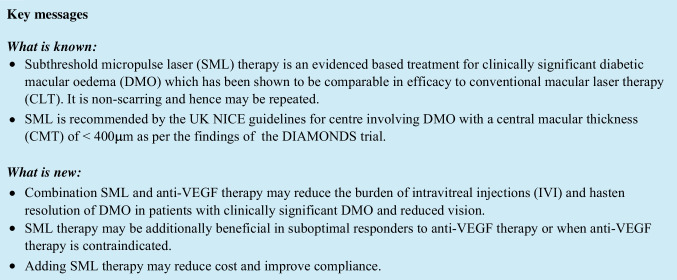


## Introduction

In 2021, the International Diabetes Federation (IDF) estimated that there were 537 million adults living with diabetes mellitus; this number is projected to rise significantly to around 783 million by 2045 [1]. With the number of adults with diabetes and clinically significant macular oedema projected to rise from an estimated 18.83 million globally in 2020 to 28.61 million by 2045, ease of training and care provision is an important consideration as the need for care increases [2]. Diabetic macular oedema (DMO) is defined as an accumulation of fluid between the outer plexiform and the inner nuclear layers of the retina and is the most common sight-threatening microvascular complication of long-standing diabetes among the working-age population (20–74 years) [3]. Diabetes results in capillary non-perfusion and retinal ischemia, creating an environment favourable to the release of vascular endothelial growth factor (VEGF) and other vitreous inflammatory factors that modulate angiogenesis and vascular permeability [4].

Currently, the gold standard of care for patients with centre involving DMO is treatment with intravitreal anti-vascular endothelial growth factor (anti-VEGF) injections [5]. Data from the Diabetic Retinopathy Clinical Research (DRCR) network’s “Protocol T trial” has shown anti-VEGF agents aflibercept, bevacizumab, and ranibizumab to be effective in reducing central macular thickness (CMT) and improving visual acuity (VA) in patients with DMO [6]. However, there are several notable limitations to the unhindered adoption of these therapies as sole agents [7]. Anti-VEGF agents aflibercept, bevacizumab, and ranibizumab have short intraocular half-lives of 10.1, 10.1, and 5.4 days respectively, resulting in high rates of non-compliance due to treatment burden and significant costs to the healthcare system [7]. Moreover, several studies have also demonstrated a significant proportion of patients with DMO have limited or absent response to intravitreal anti-VEGF monotherapy [8, 9]. Furthermore, certain clinical situations, such as a recent cerebrovascular event or pregnancy, may contraindicate the use of anti-VEGF therapy [10].

Prior to the adoption of intravitreal anti-VEGF agents, conventional laser photocoagulation therapy (CLT) was a first-line therapy in clinically significant DMO [11]. The landmark Early Treatment Diabetic Retinopathy Study (ETDRS) first reported the benefits of CLT, showing a 50% reduction in the risk of moderate visual loss over 3 years compared to no intervention for clinically significant DMO [11]. Several studies have combined anti-VEGF therapy with CLT, including the Protocol T Trial where this was the standard of care [6, 12, 13]. A reduction in the number of injections was noted in the READ-2, RESTORE, and Protocol T trials [14]. Previous studies have also demonstrated superior improvements in BCVA and CMT reduction with CLT [14]. However, the benefits of CLT with conventional laser are limited by substantial adverse effects including retinal scarring, visual field loss, subretinal fibrosis, and choroidal neovascularisation [15]. These side effects are primarily a consequence of thermal damage to the retinal pigment epithelium (RPE) leading to photocoagulation necrosis at the focal site of treatment which can rapidly expand into chorioretinal atrophy [15].

Subthreshold micropulse laser (SML) is a newer, non-destructive alternative to conventional laser therapy (CLT). SML does not cause fatal injury to the RPE, nor subsequent macular atrophy and has been shown to have comparable treatment outcomes to CLT in patients with CMT < 400 µm in the 2023 DIAMONDS trial [2]. Several studies evaluating the utility of SML as an adjunctive treatment for anti-VEGF agents have demonstrated improvements in visual outcomes and fewer injections with combined therapy [14, 16–18]. A recent meta-analysis by Chen and colleagues (2023) compared the efficacy of intravitreal anti-VEGF monotherapy with combined anti-VEGF and CLP for the treatment of DMO [7]. However, to our knowledge, there are no previous systematic reviews or meta-analyses evaluating the utility of SML as an adjunctive treatment for anti-VEGF agents. Here, we present a comprehensive systematic review of the literature and meta-analysis of the currently available data.

## Methods

### Protocol

This systematic review and meta-analysis was conducted according to the Preferred Reporting Items for Systematic Reviews and Meta-Analyses (PRISMA) framework and Cochrane Handbook guidelines [19]. The protocol for the study was registered with the PROSPERO International Register of Systematic Reviews (CRD42023450655).

### Search strategy

A comprehensive search of six databases (PubMed, Cochrane MEDLINE, Embase, Scopus, and Google Scholar) was conducted on July 03, 2023. Search terms were defined according to the PICO tool (see supplementary data). Search parameters were set for studies conducted between January 01, 2000 and July 01, 2023 and limited to full texts to ensure the most recent clinical trials were captured.

We utilised Covidence (Systematic review software, Veritas Health Innovation) to streamline the study selection process. Studies identified from database searches and snowballing were imported into Covidence. Two authors CW and JN independently screened titles and abstracts and categorised them into one of three selections—“include”, “exclude”, and “maybe”. Disagreements between reviewers were resolved with discussion and arbitrated by senior reviewers (VP and JJ) where there was a failure to reach consensus.

### Study selection and eligibility criteria

Studies were included in the review if they (1) looked at adult patients with a diagnosis of DMO treated with a combination of anti-VEGF and SLT with anti-VEGF monotherapy as a comparator; (2) papers reporting on at least one of the following outcome measures: best corrected visual acuity (BCVA), central macular thickness (CMT), or number of injections; and (3) papers reporting outcomes at baseline (0) and at least one of 6–8 months, or 12–14 or more months.

Systematic reviews, meta-analyses, other review articles, case reports, grey literature, and studies where full text was not available in English were excluded. Studies reporting upon conditions other than DMO or a combination of conditions were also excluded from the final synthesis of results.

### Data extraction

Primary (CW) and secondary (JN) reviewers independently extracted data from the included studies into a customised data extraction table. The extracted data included key identifying and demographic information including first author, year of publication, location, study design, details of intervention, baseline visual characteristics and HbA1c levels, follow-up time, and sample size. The primary outcome of interest was the number of injections at 12–14 months; secondary outcomes for extraction were CMT (microns) and BCVA (logMAR) at 6–8 months or 12–14 months or more. The extracted dataset was independently cross-checked for accuracy. Any conflicts between extracted datasets were investigated and resolved with discussion. Attempts were made to contact the authors directly for any missing data not available in supplementary information packages.

### Risk of bias assessment

Our analysis included both randomised and retrospective controlled trials; therefore, we used the Cochrane Risk of Bias 2 (RoB 2) tool for the assessment of the risk of bias in randomised studies and the Cochrane Risk of Bias in Non-randomised Studies of Interventions (ROBINS-I) tool for retrospective studies [20, 21]. Primary (CW) and secondary (JN) researchers independently completed the risk of bias assessment and independently scored each item within each domain as “low”, “moderate”, or “high/serious” for risk of bias. Disagreements in scoring were discussed and resolved with a third reviewer. The number of “low” responses for each domain of the RoB 2 and ROBINS-I was tallied, and a score was calculated to determine the overall quality of the study as follows: Low quality: (%Low) < 40%; Some concerns: (%Low) 60 to 80%; High quality: (%Low) > 80%. Studies were not excluded based on quality; however, this was considered during data analysis and in the interpretation of our findings. Results for the overall RoB and individual domains are presented in traffic light and summary plots.

### Statistical methods

#### Data preparation

Extracted data was used to calculate (1) the mean difference between the number of injections for control and intervention groups at 12–14 months, (2) subgroups according to BCVA (< 0.5 logMAR or ≥ 0.5 logMAR) and HbA1c (< 7% or ≥ 7%) at baseline, and (3) the difference of mean differences between baseline and endpoints for both control and intervention groups for BCVA and CMT.

#### Statistical analysis

Statistical analysis was performed in consultation with a medical statistician (PP). We conducted a meta-analysis of mean differences utilising a random effects model to account for inter-study variability (Der Simonian and Laird approach). We used Hedge’s g to measure the standardised difference in the means, using an estimate of the pooled standard deviation and corrected for bias.

As no papers explicitly presented a change in standard deviation (SD) from baseline, this was calculated for both control and intervention groups with the standard formula for SD change for correlated values [22, 23]. ${\text{SD}}_\text{change}=\sqrt{\text{SD}_\text{baseline}^2+\text{SD}_\text{final}^2-(2\text{Corr}\;.\;{\text{SD}}_{\mathrm{baseline}\;}.\;{\text{SD}}_\text{final})}$.

No studies were available among our included studies that reported change in SD from baseline; therefore we were unable to calculate a correlation coefficient (Corr) based on any studies reported in considerable detail. We instead hypothesised a Corr of 0.7 to impute change from baseline SDs, a reasonable “middle” value for intra-person biological variability [24]. Sensitivity analyses were conducted with a Corr of 0.6 and 0.8 to ensure the robustness of results [24].

Heterogeneity among studies was first assessed using Cochran’s *Q* test, and the *I*^2^ statistic, where a value of greater than 50% was taken to indicate statistical heterogeneity. To investigate potential sources of heterogeneity, we performed subgroup analyses based on predefined variables including mean age, sex, and sample size.

Subgroup analysis was performed according to BCVA at baseline for the number of injections. We were unable to perform subgroup analysis according to CMT or HbA1c as the baseline characteristics differed between groups. The robustness of our results was further assessed using sensitivity analyses where any outliers were detected. Data was pooled in a meta-analysis when at least four studies reported on the same outcome measures for a given time point. All meta-analyses and subgroup analyses were conducted using Stata v18.0 (Stata Corp; LLC). *p* values < 0.05 were considered statistically significant.

## Results

### Search yield

**Fig. 1:**
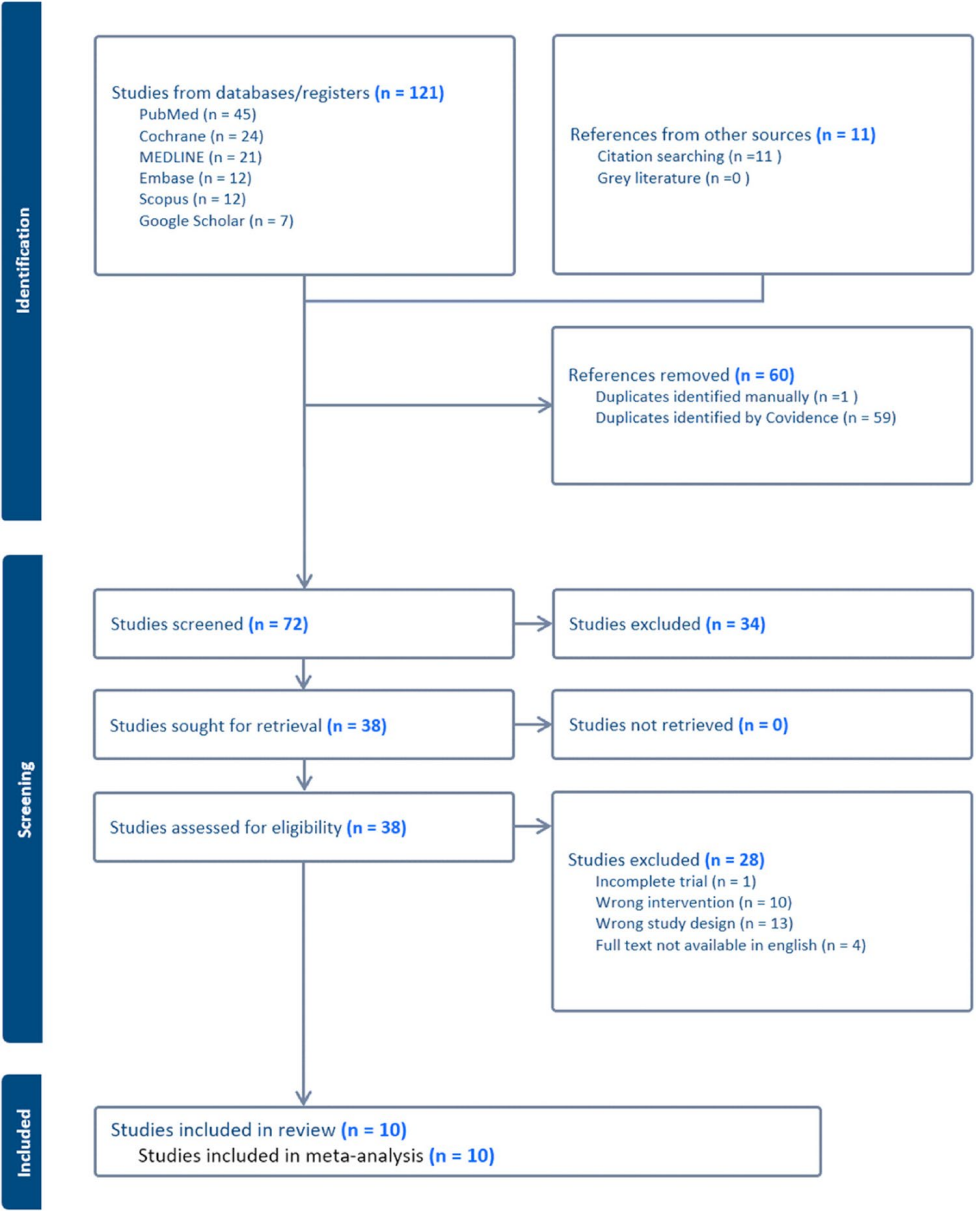
PRISMA flow diagram of study selection

A total of 132 articles were included, with 121 articles identified from the primary database search, and a further 11 records identified from snowballing of literature and reference lists. Exclusion of duplicates yielded 72 unique articles, 34 of which were further excluded following title and abstract screening. Full-text screening was undertaken for the remaining 38 articles with all papers successfully retrieved. During the full-text screening process, 28 studies were excluded; one study was incomplete at the time of review, four studies did not have the full texts available in English, 13 studies had an incompatible study design and a further ten studies did not report on combination SML therapy with anti-VEGF. The remaining ten results were selected for critical appraisal and included in the synthesis of our results (see Fig. 1). In total, our review included six randomised clinical trials and four retrospective clinical studies.

### Risk of bias assessment

**Fig. 2:**
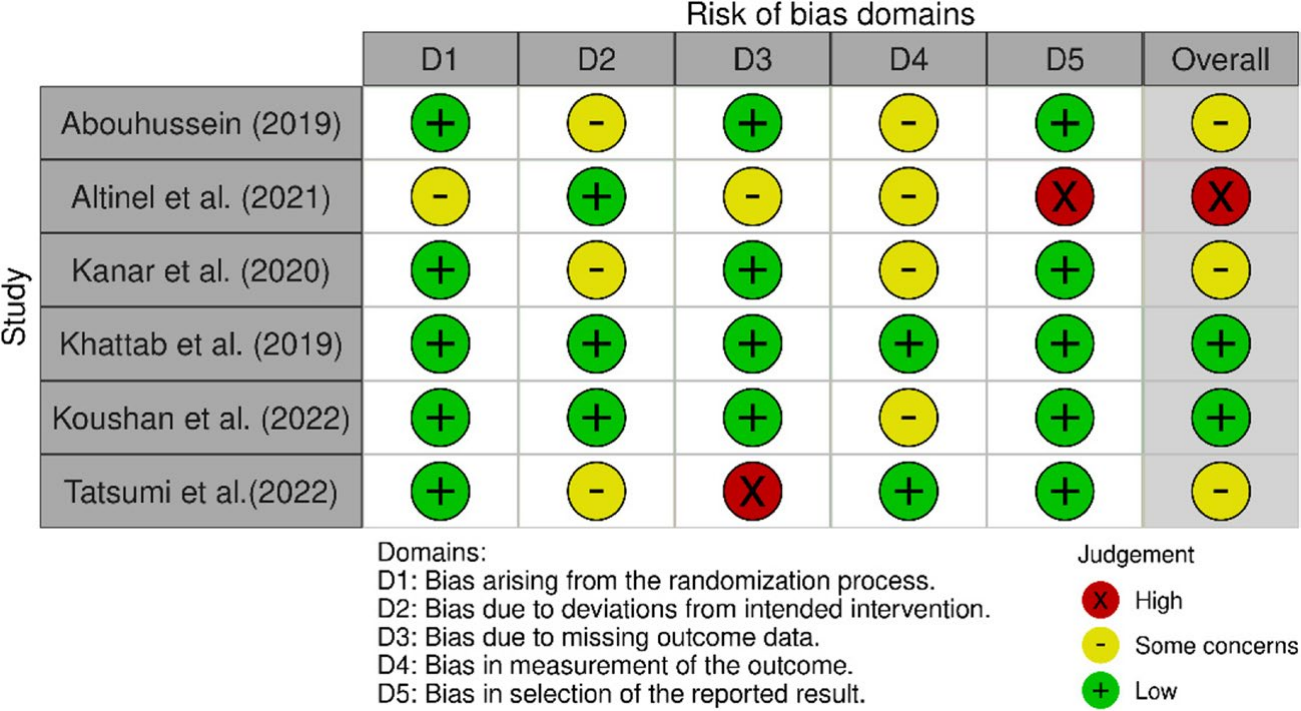
Traffic light plot of RoB assessment of RCTs (Cochrane ROB 2 tool) [32]

**Fig. 3:**
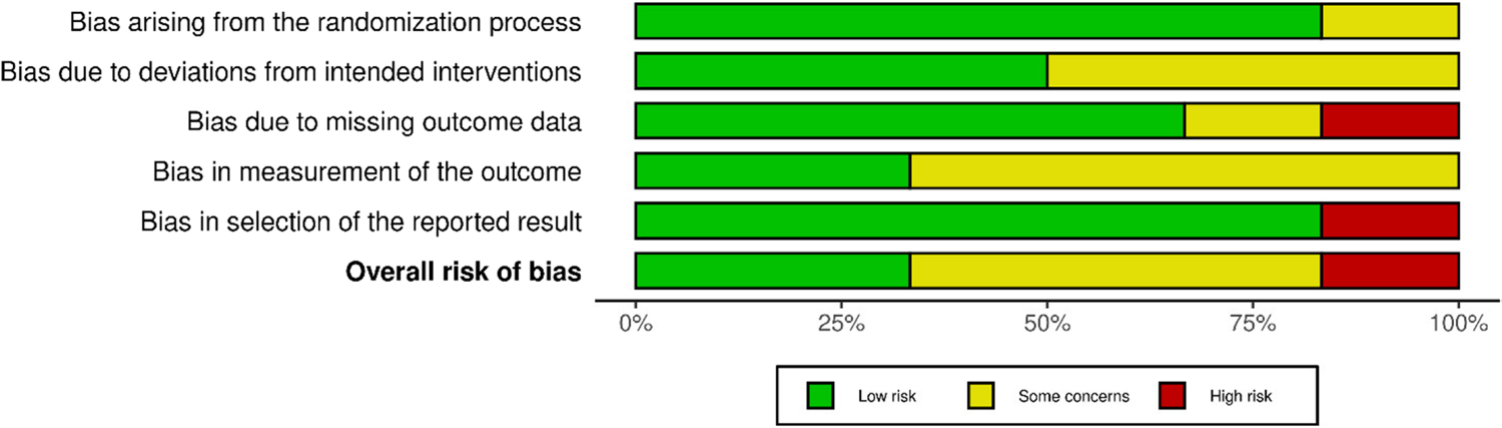
Summary plot of RoB assessment of RCTs (Cochrane ROB 2 tool) [32]

**Fig. 4:**
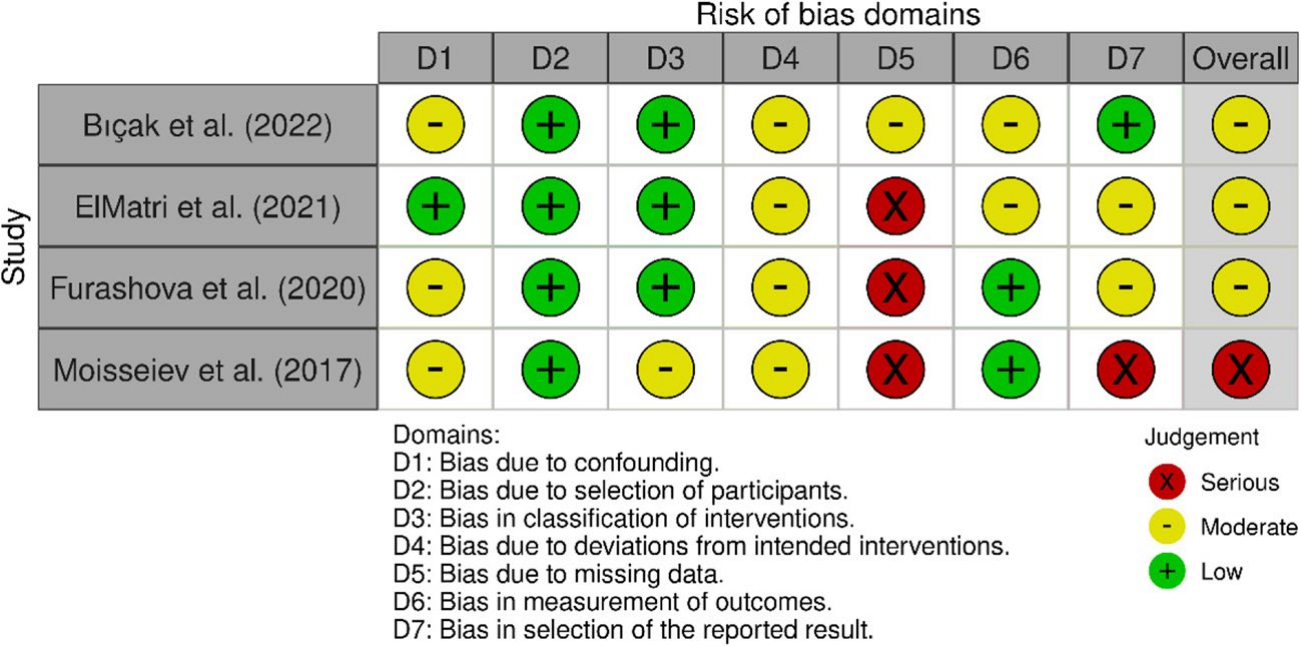
Traffic light plot of RoB assessment of retrospective studies (ROBINS I tool) [32]

**Fig. 5:**
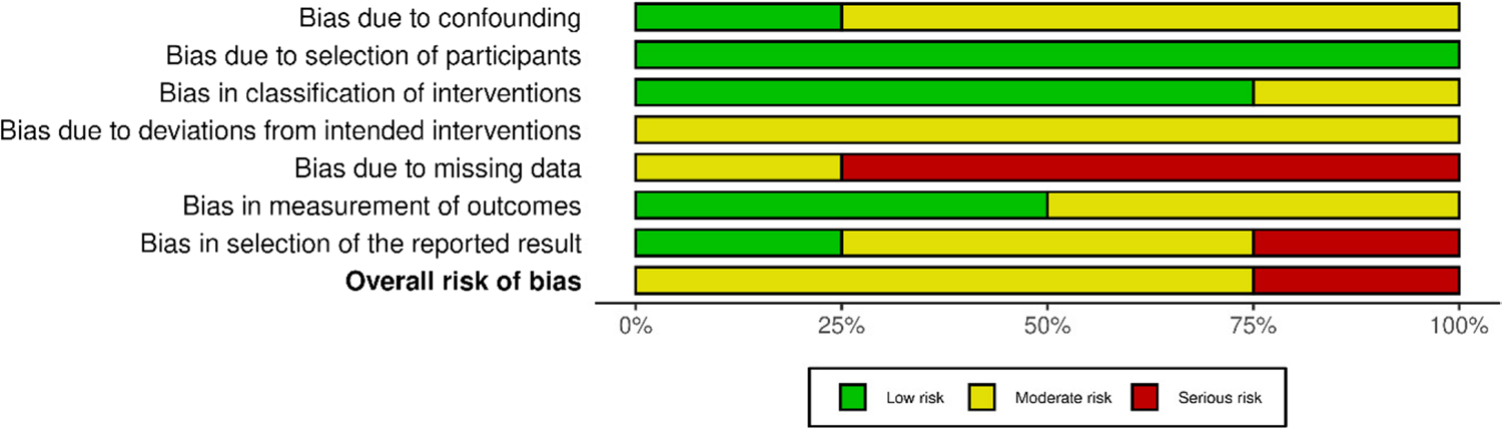
Summary plot of RoB assessment of retrospective studies (ROBINS I tool) [32]

A summary of the risk of bias assessment for randomised (Figs. 2 and 3) and retrospective trials (Figs. 4 and 5) are shown below. Overall, the risk of bias was considered “low” for two studies [25, 26], “some concerns” for five studies [14, 17, 18, 27–29], and “high” for two studies [30, 31]. Specifically, bias in the measurement of the outcomes was identified in eight studies [14, 17, 18, 26–28, 30, 31], most commonly due to unblinded outcome measurement. Two studies omitted outcome data [30, 31], while a further two papers reported only partial outcome data [17, 30]. Three studies also reported some deviations from the intended intervention protocol, unaccounted for by their analysis and not corrected for bias [18, 27, 29].

### Demographic and clinical study characteristics

Our final synthesis of data included six randomised clinical trials [18, 25–29], and four retrospective clinical trials [14, 17, 30, 31]. The characteristics of these included studies at baseline and endpoints are shown below in Table 1 and 2 respectively. A total of 563 eyes of 478 patients with DMO were captured in our synthesis, with only 85 patients contributing both eyes. Paired analysis was not considered suitable, due to the small number of studies with both eyes. We also note that the original papers including both eyes do not present paired analyses. Our final meta-analyses included 563 eyes, of which 277 eyes were assigned to IVI anti-VEGF monotherapy, and 286 eyes were assigned to IVI anti-VEGF with SML combination. Sample sizes of the included studies were generally small and varied from 19 to 97 participants. Overall, the mean age of participants across all studies was 61.25 years. There was a slight overrepresentation of male eyes in both monotherapy (176 M:147 F) and combination (172 M:141 F) groups.

**Table 1:** Baseline characteristics of included studies

Study		Population						Exposure	
Author (year)	Country	Eyes (*n*)		Mean age (year)		Gender (*n*)		anti-VEGF agent	Laser wavelength (nm)
		Control	Intervention	Control	Intervention	Control (m/f)	Intervention (m/f)		
Altınel et al. (2021) [30]	Turkey	40	40	59.83 ± 7.7	60.55 ± 7.23	22/18	23/17	Bevacizumab	577
Bıçak et al. (2022) [14]	Turkey	45	52	61.6 ± 6.7	62.4 ± 7.6	20/25	28/24	Ranibizumab	577
ElMatri et al. (2021) [17]	Tunisia	49	49	61.3 ± 4.11	67.7 ± 5.23	20/11	19/13	Bevacizumab	577
Furashova et al. (2020) [28]	Germany	9	10	70.78 ± 8.96	70.70 ± 7.60	6/3	8/2	Ranibizumab	810
Kanar et al. (2020) [18]	Turkey	28	28	62.64 ± 9.03	62.64 ± 9.03	32/24	32/24	Aflibercept	577
Khattab et al. (2019) [25]	Kuwait	27	27	55.7 ± 3.4	59.4 ± 4.3	16/11	11/16	Aflibercept	577
Koushan et al. (2022) [26]	Canada	15	15	58.8 ± 9.28	59.8 ± 9.47	7/8	10/5	Aflibercept	532
Tatsumi et al. (2022) [29]	Japan	25	26	69.3 ± 7.4	66.9 ± 9.4	16/8	11/14	Aflibercept	577
Abouhussein et al. (2019) [27]	Egypt	20	20	59.5 ± 4.3	60.4 ± 4.2	8/12	9/11	Aflibercept	577
Moisseiev et al. (2017) [31]	Israel/USA	19	19	63.3 ± 8.9	65.3 ± 9.8	13/6	12/7	Ranibizumab	577

Study		Exposure		Outcomes					
Author (year)	Country	Dosage and timing		CMT		BCVA		HbA1c %	
		IVI anti-VEGF only	IVI anti-VEGF + micropulse adjunct	CMT (cont)	CMT (int)	BCVA (cont)	BCVA (int)	HbA1c (cont)	HbA1c (int)
Altınel et al. (2021) [30]	Turkey	3 × monthly IVI 1.25 mg/0.05 mL IVI + IVI PRN	3 × monthly IVI 1.25 mg/0.05 mL IVI + SML 4 weeks post loading dose if CMT < 400 um	384.68 ± 64.11	379.2 ± 70.25	0.39 ± 0.23	0.38 ± 0.21	6.89 ± 0.61	6.94 ± 0.53
Bıçak et al. (2022) [14]	Turkey	3 × monthly IVI 0.5 mg/0.05 mL + monthly IVI PRN	3 × monthly IVI 0.5 mg/0.05 mL + SML 4 weeks post IVI loading once ONLY	406 ± 130.4	426.6 ± 96.9	0.41 ± 0.25	0.43 ± 0.23	6.85 ± 0.59	6.91 ± 0.54
ElMatri et al. (2021) [17]	Tunisia	3 × monthly IVI 1.25 mg/ 0.05 mL + IVI PRN 4 weekly	3 × monthly IVI 1.25 mg/ 0.05 mL + SML within 1 week of 3rd injection	359.9 ± 22.9	479.1 ± 14.3	0.60 ± 0.42	0.69 ± 0.35	7.60 ± 062%	7.70 ± 0.81
Furashova et al. (2020) [28]	Germany	3 × monthly IVI 0.5 mg/0.05 mL + IVI PRN	3 × monthly IVI 0.5 mg/0.05 mL +	485 ± 170	434 ± 118	0.78 ± 0.2	0.68 ± 0.2	7.54 ± 1.51	6.80 ± 0.85
Kanar et al. (2020) [18]	Turkey	3 × monthly IVI 2 mg/0.05 mL + IVI PRN	3 × monthly IVI 2 mg/0.05 mL + SML at 1 month if CMT decreased < 450 um if > 450 um a second course of IVI + SML applied at 8 weeks	451.28 ± 44.85	466.07 ± 71.79	0.94 ± 0.1	0.90 ± 0.09	8.02 ± 2.43	7.97 ± 2.47
Khattab et al. (2019) [25]	Kuwait	3 × monthly IVI of 2 mg/0.05 mL + IVI PRN suspended when CMT reached 250 um or less	3 × monthly IVI of 2 mg/0.05 mL + SML within 1 week of the third injection	462 ± 32.2	457.1 ± 22.6	1.07 ± 0.36	1.00 ± 0.35	NA	NA
Koushan et al. (2022) [26]	Canada	1 × IVI 2.0 mg/0.05 mL + sham laser on the same day + 4 weekly sham laser	1 × IVI 2.0 mg/0.05 mL + SML on the same day + SML 4 weekly`	433.4 ± 103.5	457.8 ± 92.8	0.38 ± 0.14	0.36 ± 0.21	NA	NA
Tatsumi et al. (2022) [29]	Japan	3 × monthly IVI of 2.0 mg/mL IVI + IVI PRN	3 × monthly IVI of 2.0 mg/mL IVI + SML within 4 weeks of 3rd IVI + IVI PRN	442.8 ± 91.3	476.4 ± 136.1	0.37 ± 0.24	0.48 ± 0.32	NA	NA
Abouhussein et al. (2019) [27]	Egypt	3 × monthly IVI of 2 mg/0.05 mL loading + PRN IVI	3 monthly IVI of 2 mg/0.05 mL loading + SML session 1 month later + monthly PRN IVI	457.9 ± 82.2	469.6 ± 78	0.7 ± 0.24	0.26 ± 0.16	8.2 ± 1.2	8.7 ± 1.1
Moisseiev et al. (2017) [31]	Israel/USA	3 × monthly 0.3 mg/0.05 mL	3 × monthly 0.3 mg/0.05 mL + SML at 2 months post final IVI	408.4 ± 104.2	316.8 ± 91.5	0.41 ± 0.13	0.29 ± 0.12	NA	NA

*anti-VEGF*, anti-vascular endothelial growth factor; *BCVA*, best corrected visual acuity; *CMT*, central macular thickness change; *CLT*, combination laser therapy; *SML*, subthreshold micropulse laser; *IVI*, intravitreal injection; *PRN*, pro re nata; *NA*, not applicable; *HbA1c*, glycated haemoglobin

**Table 2:** Characteristics of included studies at endpoints (6–8 months and 12–14 months)

Study	Population						Outcomes			
Author (year)	Eyes (*n*)		Mean age (year)		Gender (*n*)		CMT (6–8 m)		BVCA (6–8 m)	
	Control	Intervention	Control	Intervention	Control (m/f)	Intervention (m/f)	CMT (cont)	CMT (int)	BCVA (cont)	BCVA (int)
Akhlaghi et al. (2019) [16]	21	21	60.86 ± 8.57	60.86 ± 8.57 years	10/11	10/11	NA	NA	NA	NA
Altınel et al. (2021) [30]	40	40	59.83 ± 7.7	60.55 ± 7.23	22/18	23/17	337.13 ± 103.64	300.45 ± 44.29	0.33 ± 0.28	0.27 ± 0.16
Bıçak et al. (2022) [14]	45	52	61.6 ± 6.7	62.4 ± 7.6	20/25	28/24	NA	NA	NA	NA
Cuervo-Lozano et al. (2018) [34]	10	10	56.30 ± 7.78	59.60 ± 5.50	2/4	3/3	NA	NA	NA	NA
ElMatri et al. (2021)[17]	49	49	61.3 ± 4.11	67.7 ± 5.23	20/11	19/13	NA	353.2 ± 17.2	NA	0.64 ± 0.33
Furashova et al. (2020) [28]	9	10	70.78 ± 8.96	70.70 ± 7.60	6/3	8/2	NA	NA	NA	NA
Kanar et al. (2020) [18]	28	28	62.64 ± 9.03	62.64 ± 9.03	32/24	32/24	387.92 ± 47.71	377.3 ± 45.61	0.26 ± 0.09	0.23 ± 0.1
Khattab et al. (2019) [25]	27	27	55.7 ± 3.4	59.4 ± 4.3	16/11	11/16	295.7 ± 53.1	295.1 ± 29.6	0.85 ± 0.40	0.77 ± 0.31
Koushan et al. (2022) [25]	15	15	58.8 ± 9.28	59.8 ± 9.47	7/8	10/5	309.3 ± 52	302 ± 61.5	0.32 ± 0.19	0.21 ± 0.13
Tatsumi et al.(2022) [29]	25	26	69.3 ± 7.4	66.9 ± 9.4	16/8	11/14	NA	NA	NA	NA
Abouhussein et al. (2019) [27]	20	20	59.5 ± 4.3	60.4 ± 4.2	8/12	9/11	292.3 ± 25.3	288.1 ± 29.6	0.26 ± 0.09	0.26 ± 0.18
Moisseiev et al. (2017) [31]	19	19	63.3 ± 8.9	65.3 ± 9.8	13/6	12/7	NA	NA	NA	NA

Study	Outcomes									
Author (year)	CMT (12–14 m)		BCVA (12–14 m)		Number of injections (mean ± SD)		Subgroup			
	CMT (cont)	CMT (int)	BCVA (cont)	BCVA (int)	Control	Intervention	BVCA	HbA1c		
Akhlaghi et al. (2019) [16]	NA	NA	NA	NA	NA	NA	2	NA		
Altınel et al. (2021) [30]	325.8 ± 92.67	292.64 ± 57.22	0.32 ± 0.24	0.25 ± 0.17	5.65 ± 1.51	4.38 ± 0.81	1	1		
Bıçak et al. (2022) [14]	304.25 ± 45.53	311.31 ± 35.77	0.69 ± 0.17	0.71 ± 0.06	4.19 ± 1.01	5.53 ± 1.14	1	1		
Cuervo-Lozano et al. (2018) [34]	NA	NA	NA	NA	NA	NA	1	NA		
ElMatri et al. (2021)[17]	305.9 ± 0.38	289.6 ± 15.0	0.49 ± 0.32	0.50 ± 0.37	7.2 ± 1.3	4.1 ± 1.5	2	2		
Furashova et al. (2020) [28]	422.5 ± 67.57	316.62 ± 82.71	NA	NA	6.9 ± 0.83	3.9 ± 0.46	2	2		
Kanar et al. (2020) [18]	328.8 ± 49.69	312 ± 39.29	0.2 ± 0.1	0.17 ± 0.06	5.39 ± 1.54	3.21 ± 0.41	2	2		
Khattab et al. (2019) [25]	279.3 ± 52.7	274 ± 26.9	0.75 ± 0.35	0.65 ± 0.24	7.3 ± 1.1	4.1 ± 1.1	2	NA		
Koushan et al. (2022) [25]	288.3 ± 38.2	289.5 ± 42.7	0.32 ± 0.22	0.36 ± 0.21	8.5 ± 3.3	7.9 ± 3.6	1	NA		
Tatsumi et al.(2022) [29]	347.3 ± 58.9	344.7 ± 73.1	0.22 ± 0.17	0.28 ± 0.22	4.3 ± 1.36	4.48 ± 1.34	1	NA		
Abouhussein et al. (2019) [27]	290.5 ± 49.5	288.5 ± 46.4	0.24 ± 0.22	0.2 ± 0.21	5.4 ± 1.7	4.5 ± 1.4	2	NA		
Moisseiev et al. (2017) [31]	335.9 ± 69.8	282.6 ± 59.1	0.39 ± 0.15	0.24 ± 0.17	5.6 ± 2.1	1.7 ± 2.3	1	NA		

*anti-VEGF*, anti-vascular endothelial growth factor; *BCVA*, best corrected visual acuity; *CMT*, central macular thickness change; *SML*, subthreshold micropulse laser; *IVI*, intravitreal injection; *PRN*, pro re nata; *NA*, not applicable; *HbA1c*, glycated haemoglobin

Aflibercept was the most commonly used anti-VEGF agent among the included studies, used in five studies [18, 25–27, 29] followed by ranibizumab which was used in three studies [14, 28, 31] and bevacizumab, used in two studies [17, 30].

In terms of laser characteristics, eight of the included studies used a wavelength of 577 nm [14, 17, 18, 25, 27, 29, 30] which is the standard commercial emphasis. Only Furashova et al. used 810-nm wavelength laser which has a larger therapeutic range [28, 33].

### Effect of combination therapy on the number of injections

**Fig. 6:**
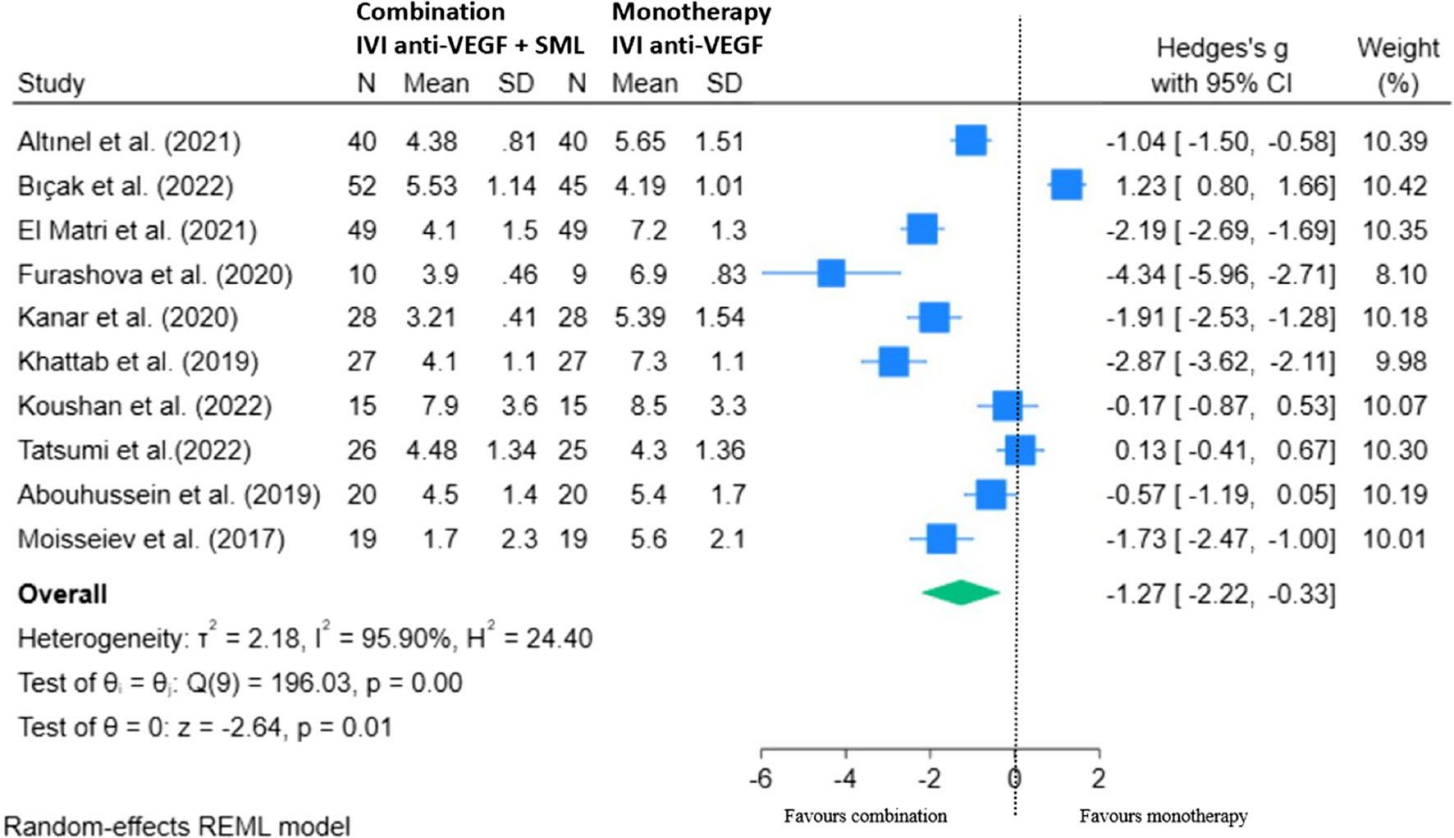
REML of required number of injections with combination (IVI anti-VEGF + SML) versus monotherapy (control) at 12–14 months

Ten studies met the criteria for inclusion in our meta-analysis to determine the effect of combination IVI anti-VEGF with SML adjunct upon the number of injections at 12 months (Fig. 6). Overall, our results showed the number of injections required was significantly lower with combination therapy. Patients with DMO who were treated with SML adjunct required greater than one injection (− 1.27) less over 12–14 months compared with those treated with anti-VEGF monotherapy, mean difference: − 1.27 (95% CI: − 2.22, − 0.33; *p* < 0.05).

#### Effect of baseline BVCA on the number of injections

**Fig. 7:**
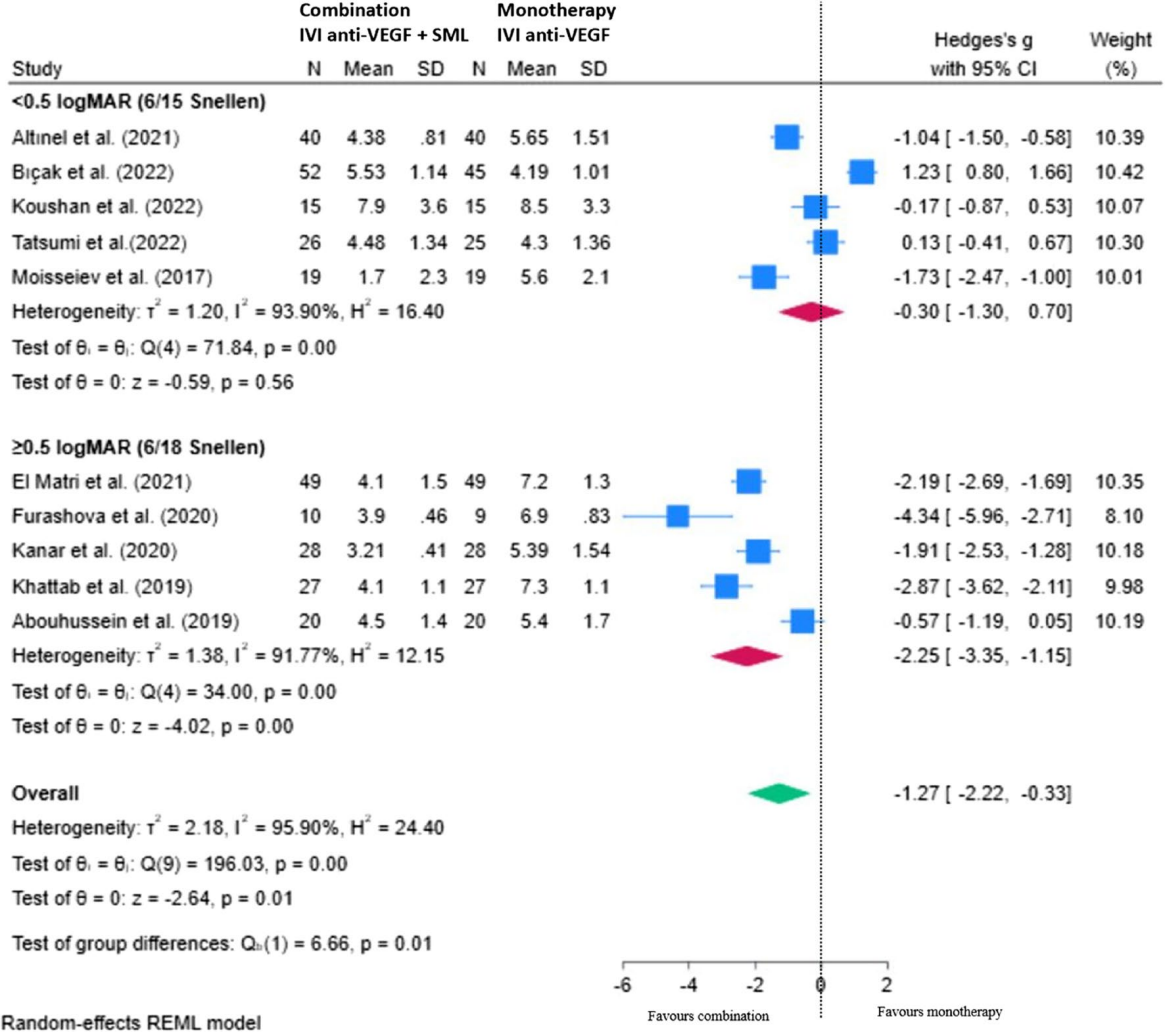
Subgroup analysis showing the effect of baseline BCVA on NOI with combination (IVI anti-VEGF + SML) versus monotherapy (control) at 12–14 months

Subgroup analysis was performed to investigate the effect of baseline visual acuity on the number of injections (NOI) required at 12 months (Fig. 7). Studies were classified into two groups for subgroup analysis according to baseline visual acuity (6/15 Snellen or better, or 6/18 Snellen or worse). Patients with poorer visual acuity (6/18 Snellen or worse at baseline), showed a greater benefit from combination therapy in terms of number of injections, with greater than two fewer injections over 12–14 months compared to monotherapy comparator, mean difference − 2.25 (95% CI; − 3.35, − 1.15; *p* < 0.05). Patients with a visual acuity of 6/15 Snellen or better at baseline did not show a statistically significant reduction in the number of injections compared to the monotherapy comparator, mean difference − 0.30 (95% CI; − 1.30, 0.70; *p* > 0.05). Overall, patients with a poorer baseline visual acuity of 6/18 Snellen or worse benefited from a greater, statistically significant injection-sparing effect from the combination SML therapy compared with those with a higher baseline visual acuity of 6/15 Snellen or better.

### Effect of IVI anti-VEGF monotherapy versus micropulse (SML) combination on BCVA

#### BCVA at 6–8 months

**Fig. 8:**
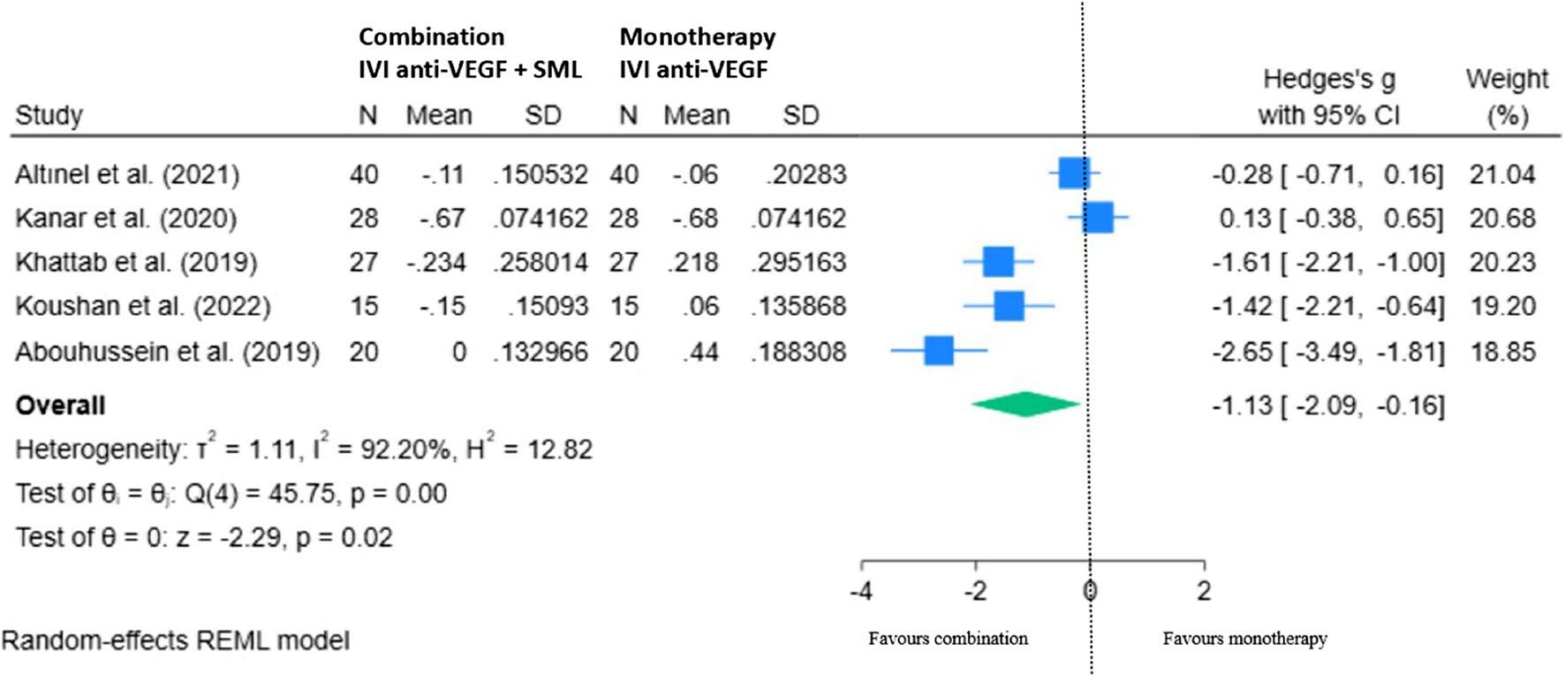
REML of change in BCVA (logMAR) for combination (IVI anti-VEGF + SML) minus monotherapy (IVI anti-VEGF) at 6–8 months

A random effects model (REML) of five RCTs shows the mean change in BCVA with combination treatment minus monotherapy at 6 to 8 months (Fig. 8). Overall, there was a statistically significant overall improvement in BCVA (logMAR) in the combination group compared to monotherapy with anti-VEGF therapy alone, mean difference of − 1.13 (95% CI − 2.09, − 0.16; *p* < 0.05).

#### BCVA at 12 – 14 months

**Fig. 9:**
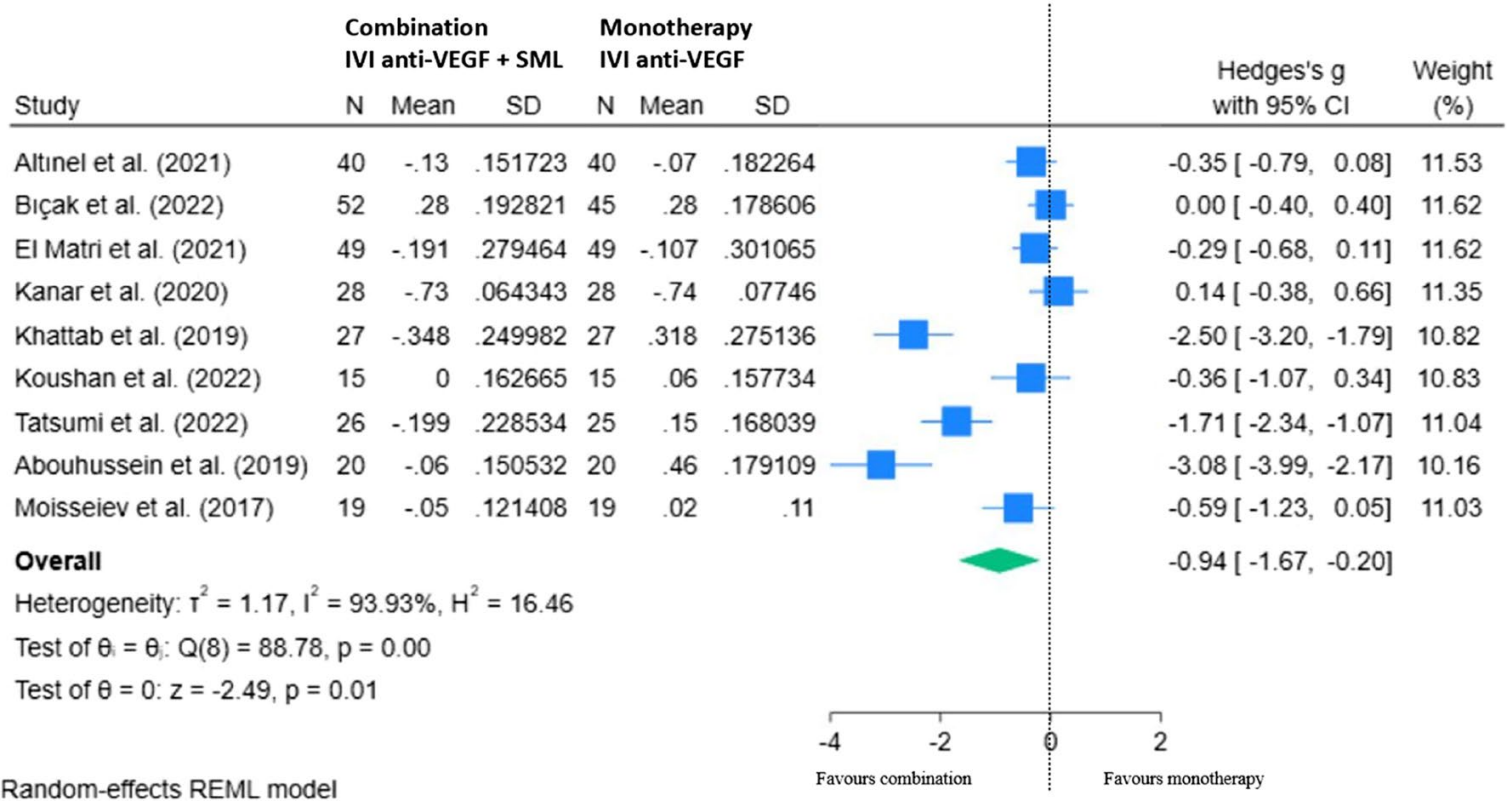
REML of change in BCVA (logMAR) for combination (IVI anti-VEGF + SML) minus monotherapy (IVI anti-VEGF) at 12–14 months

Nine RCTs are included in our REML showing the mean change in BCVA with combination therapy minus monotherapy at 12–14 months from baseline (Fig. 9). Overall, there was a statistically significant overall improvement in BCVA with SML adjunct compared to anti-VEGF therapy monotherapy, mean difference of − 0.94 (95% CI − 1.67, − 0.20; *p* < 0.05).

### Effect of IVI anti-VEGF therapy with SML combination versus anti-VEGF therapy alone on CMT

We were unable to stratify studies according to CMT at baseline to perform a subgroup analysis as baseline characteristics for CMT between combination and monotherapy comparators were not homogeneous.

#### CMT 6 – 8 months combination therapy minus monotherapy

**Fig. 10:**
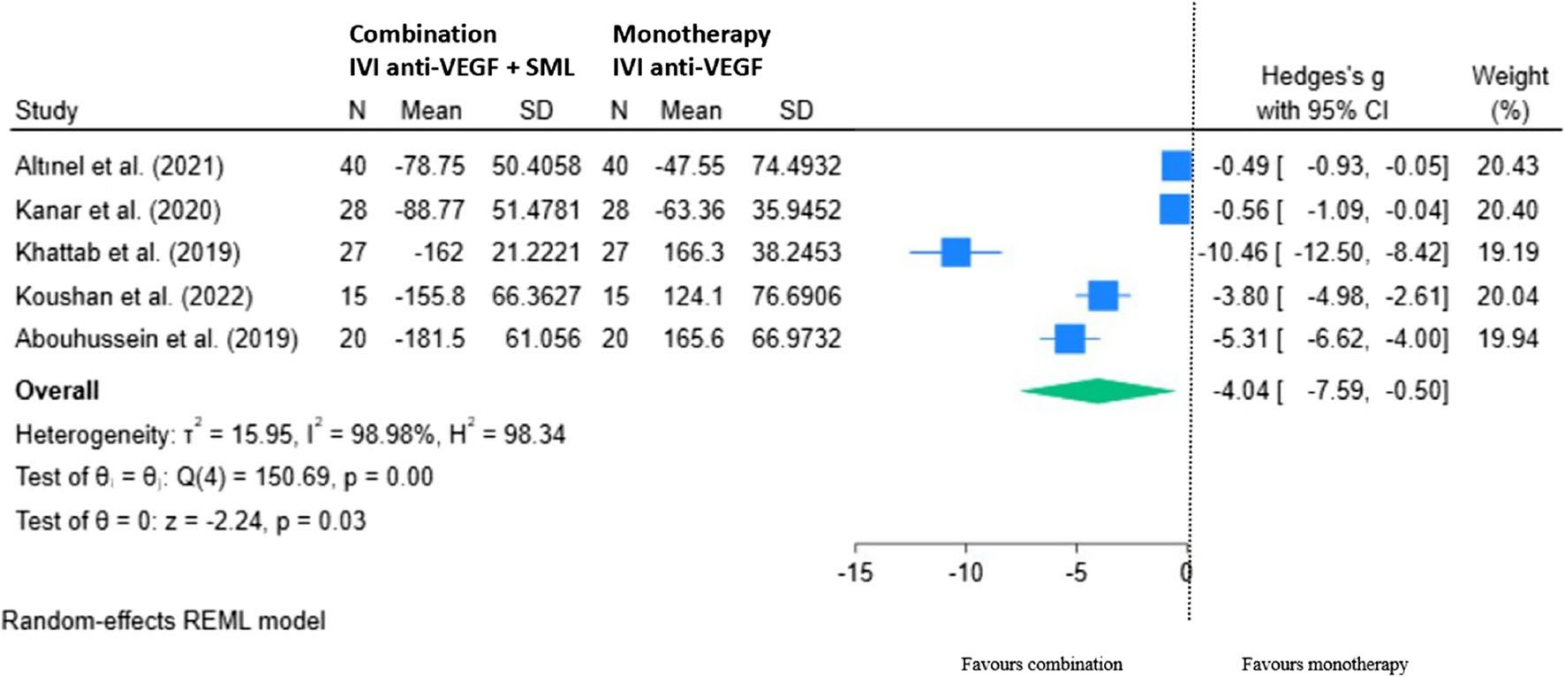
REML of change in CMT (µm) for combination (IVI anti-VEGF + SML) minus monotherapy (IVI anti-VEGF) at 6–8 months

Five RCTs are included in our meta-analysis of mean change in CMT with combination monotherapy at 6 to 8 months from baseline (Fig. 10). Overall, there was a statistically significant improvement in CMT with SML adjunct compared to anti-VEGF therapy alone; mean difference of − 4.04 (95% CI − 7.59, − 0.50; *p* < 0.05).

#### CMT 12 – 14 months

**Fig. 11:**
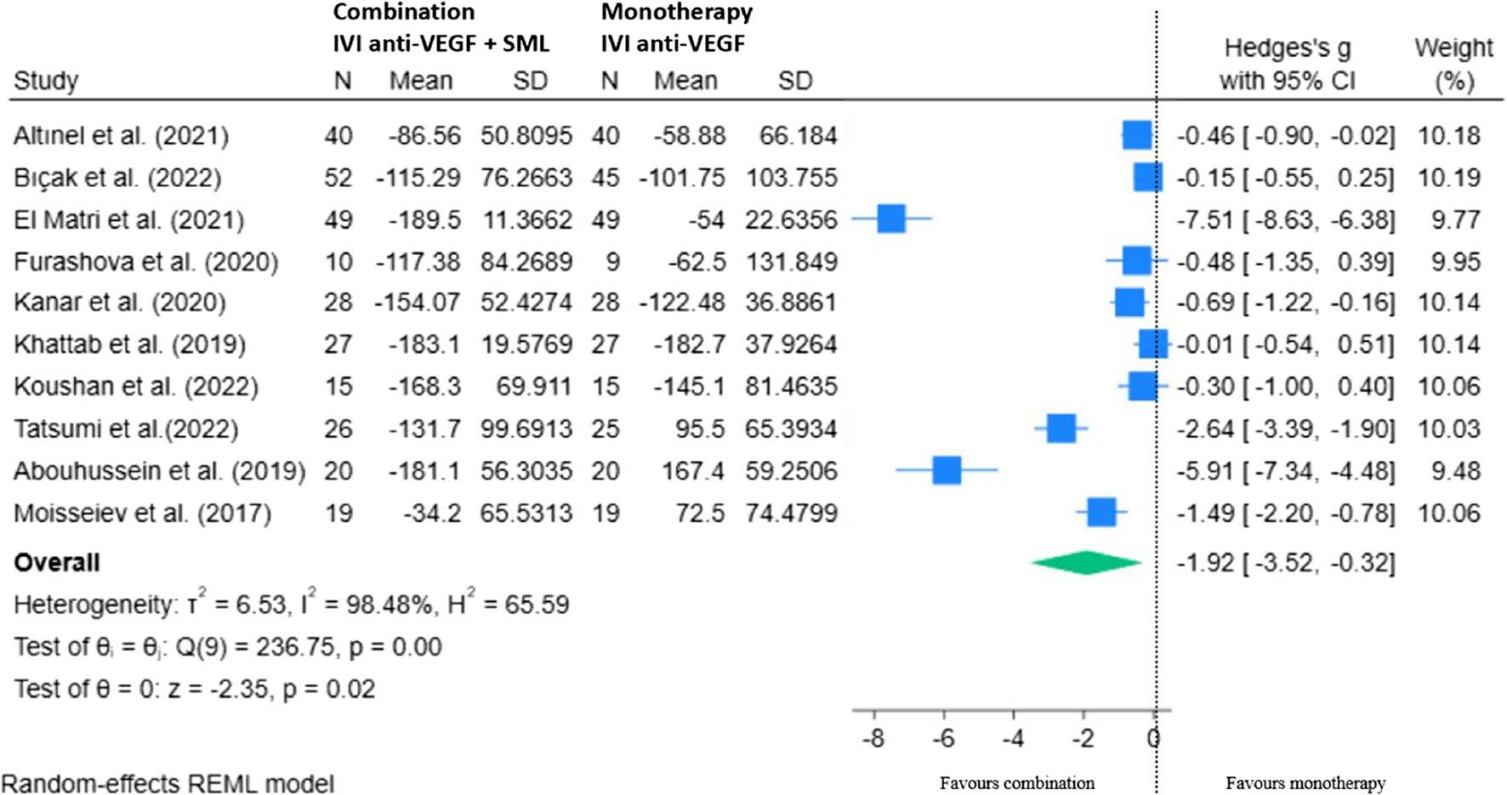
REML of change in CMT (µm) for combination (IVI anti-VEGF + SML) minus monotherapy (IVI anti-VEGF) at 12–14 months

Ten RCTs were included in our final REML showing the mean change in CMT with combination therapy minus monotherapy at 12 to 14 months from baseline (Fig. 11). There was a statistically significant overall improvement in CMT in the combination group compared to anti-VEGF therapy alone, mean difference of − 1.92 (95% CI − 3.52, − 0.32; *p* < 0.05).

### Sensitivity analyses

**Fig. 12:**
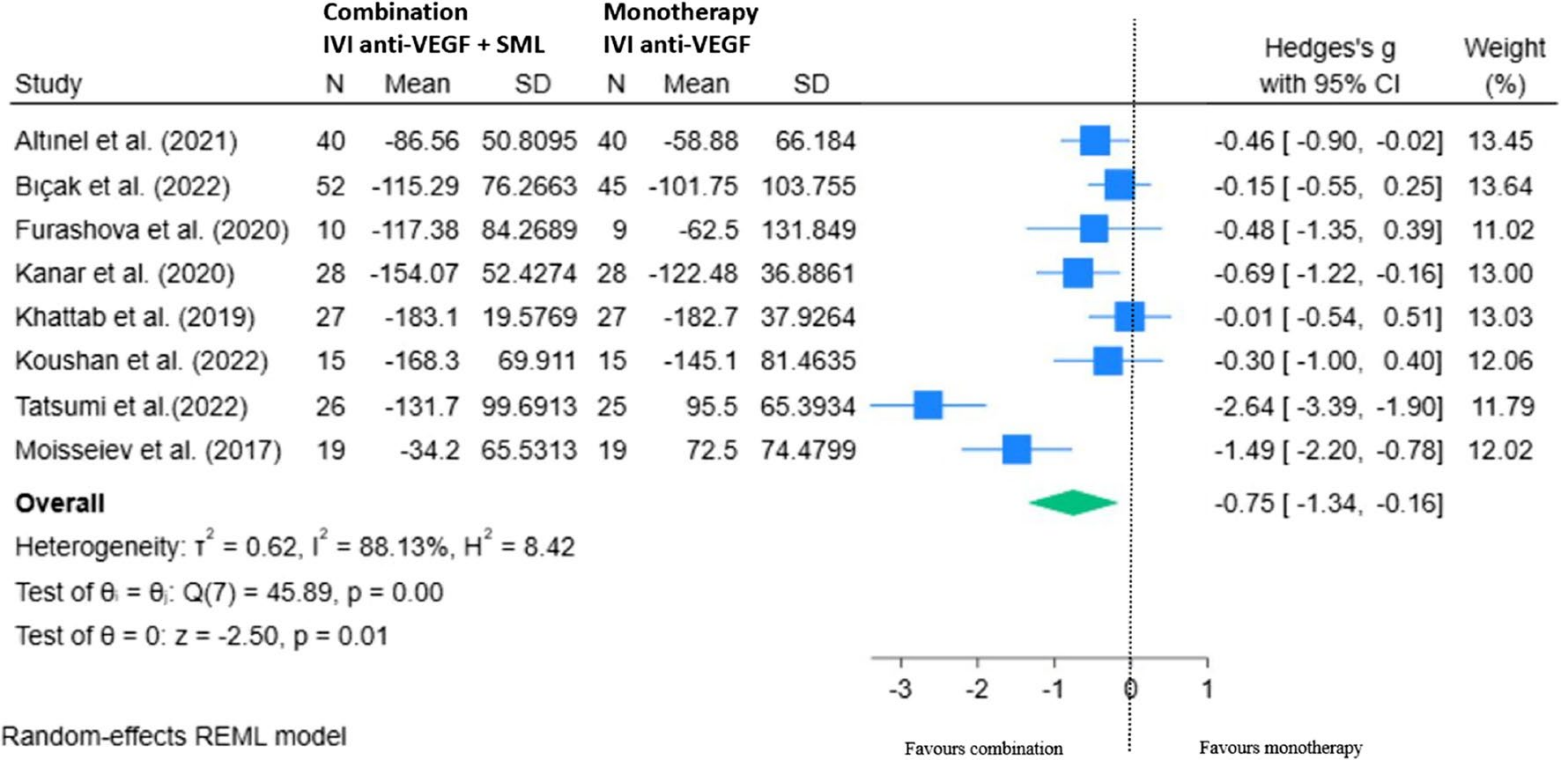
REML showing sensitivity of change in CMT (µm) at 12–14 months for combination (IVI anti-VEGF + SML) minus monotherapy (IVI anti-VEGF) with outliers excluded

Sensitivity analysis did not alter the pooled estimate of any of our meta-analyses significantly. Visually, two outliers were noted in the REML for CMT at 12 – 14 months (Fig. 11). A REML for the remaining eight studies shows sustained significance at the 95% CI following the exclusion of the Abouhussein et al. and Elmatri et al. datasets (Fig. 12), mean difference of − 0.75 (95% CI − 1.34, − 0.16).

Sensitivity analyses were also conducted using 0.6 and 0.8 Corr which did not demonstrate any significant changes to results at the 95% CI for any of the included REMLs (see supplementary files).

## Discussion

### Summary of findings

In this paper, we present a comprehensive systematic review and meta-analysis of the currently available literature comparing the efficacy of anti-VEGF monotherapy against combined anti-VEGF therapy with SML adjunct in the management of DMO. To our knowledge, this is the first such study to do this comprehensively. In total, our study utilised information from 625 eyes of 555 patients, including both eyes in 65 patients. Our findings demonstrate a significant reduction in IVI anti-VEGF injection burden with combination treatment compared to IVI anti-VEGF monotherapy. We also report improved BCVA and a reduction in CMT at 6–8 and 12–14 months from baseline with combination treatment to the IVI anti-VEGF monotherapy comparator.

The implication is that the addition of SML therapy in this context hastens the resolution of macular oedema in the first year of therapy. It is the resolution of macular oedema that will prevent vision loss in the medium term. Moreover, the addition of SML reduces the burden of intravitreal injections and hence the number of visits. It is technically possible to perform SML on the same day as IVI therapy.

Approximately one-third of patients are non-responders to anti-VEGF therapy alone [13, 33]. According to a post-hoc analysis by Bressler of the Protocol T trial, DMO can persist after anti-VEGF injection in 31.6 – 65.6% of diabetic patients [35]. Wells et al. showed a high recurrence rate after DMO resolves which often requires repeat injections of anti-VEGF [36].

Several studies have demonstrated the superiority of SML over conventional therapy (references 24 – 29 per Moisseiev et al. in terms of improving macular sensitivity for example on microperimetry and improving visual function [31]. Micropulse laser is used as first line for centre-involving and non-centre involving DMO in the UK, for patients routinely with a CMT of < 400 μm [2].

### Number of injections

Frequent injections are a requirement of all currently available anti-VEGF agents as an inherent function of their short intraocular half-lives [37, 38]. Repeated injections are associated with cumulative ocular and systemic risks, which include endophthalmitis, tractional-rhegmatogenous retinal detachment, ocular inflammation, and thromboembolic events [27, 39, 40] SML has been demonstrated to be safe with no visible scarring and none of the ocular or systemic risks of intravitreal injections. Moreover, it has an additional role in certain clinical situations such as stroke and during pregnancy where anti-VEGF therapy may be contraindicated [41].

### Visual acuity

In the study by El Matri, participants were classified as “good responders” and “poor responders” based on improvement in BCVA at 12 months [17]. The proportion of “good responders” reached 93.8% at 32 and 48 weeks [17]. The benefits of SML may not be seen for several months which is consistent with our findings [17]. SML may result in fewer DMO recurrences, and laser may have a longer-lasting benefit than anti-VEGF therapy alone [10, 42].

### Central macular thickness

Micropulse laser is more effective in patients with initial CMT of < 450 um [43–45]. Mansouri et al. showed that MPL has better efficacy in moderate DMO (CMT < 400 um). Citrik further suggested that MPL produces significant improvement in BCVA and reduction in CMT when used in eyes with CMT of 300 um or less. Inadequate treatment with SML has been reported in severe DMO, and the reason for this is not completely clear [31, 44]. One possible explanation postulated by Altinel et al. is that severe oedema may change the distribution of laser energy throughout the retina [30].

### Contrast sensitivity

Contrast sensitivity is not a commonly assessed parameter in the studies and could be affected even in patients with preserved VA. It could be considered a useful parameter as part of a functional visual assessment in DMO. Vujosevic demonstrated significantly increased retinal sensitivity on microperimetry following SML; with no detectable laser scar at 12-month follow-up, similar findings have been reported by Chhablani et al. [41, 46]. Scholz demonstrated the superiority of SML compared to conventional laser in improving anatomical and functional vision [10].

### Type of anti-VEGF agent

Wells (2016) examined the effectiveness of the different anti-VEGF therapies (aflibercept, bevacizumab, and ranibizumab) and found that changes in BCVA are highly dependent on pre-treatment VA, and aflibercept appeared to be more effective when initial visual acuity is poorer [36]. This is further supported by congruent findings from the protocol T trial. Previous studies have shown conbercept, to be a suitable alternative which has multiple targets with strong affinity and long action time (Liu, 2022). When combined with SML, this study demonstrated a higher total response rate (90.91% versus 67.76%; *p* < 0.05) and lower injection frequency in the combination group (*p* < 0.05) [47, 48]. Moreover, the combination group demonstrated higher BCVA and lower CMT after 6 months of treatment (*p* < 0.05); in addition to lower VF grey value and mean VFD, higher 30°-VF average light threshold sensitivity 1 month post treatment, all reached statistical significance [47].

### Type of laser

After demonstrating the superiority of high-density subthreshold micropulse laser (SML) in improving visual acuity and reducing CMT when compared to a standard threshold and low-density SML (2011), Vujosevic subsequently showed that both yellow (577 nm) and infrared (810 nm) lasers are safe in mild centre involving DMO with no significant difference between the two [45]. Chang confirmed that the two are equally effective, although 810-nm MPL appears to have a wider therapeutic range and therefore safety margin [49]. No study has compared their efficacy, particularly in combination with anti-VEGF treatment. Sramek proposed that 810 nm of diode laser supports the recovery of cells, especially those of the retinal pigment epithelium [50]. Midena also showed additional anti-inflammatory benefits of SML [51]. Laser comes at a fraction of the cost of injections and is more readily available, with greater graduate ophthalmologist training availability should increase [2, 10].

### Limitations

Most studies in our review are limited by small sample sizes with relatively short periods of follow-up. Many are retrospective studies [14, 17, 31, 47], where there is a lack of pre-defined criteria for the use of SML, which were generally performed at the treating clinician’s discretion. The real world setting may be different to the randomised trials where monthly injections were administered. Clinical practice may be more akin to these retrospective studies where there are lower injection rates. Measures such as microperimetry and contrast sensitivities were not always available. Most studies used a combination of ocular coherence tomography (OCT) and VA assessments to guide retreatment. HbA1C was not uniformly recorded with diabetic therapy and hence sub analysis was not possible.

## Conclusions

Combination anti-VEGF therapy with SML adjunct may reduce the burden of intravitreal anti-VEGF therapy, particularly in patients with poorer baseline visual acuity. It may also improve the BCVA and hasten the resolution of macular oedema as measured by CMT. Moreover, it may be additionally beneficial in cohorts of patients who are suboptimal responders or in whom anti-VEGF therapy is contraindicated.

## Data Availability

The original contributions presented in the study are included in the article and supplementary material. Further inquiries can be directed to the corresponding author.

## Abbreviations

anti-VEGF: Anti-vascular endothelial growth factor
BCVA: Best corrected visual acuity
CMT: Central macular thickness change
CLT: Combination laser therapy
CLP: Conventional laser photocoagulation
DMO: Diabetic macular oedema
DR: Diabetic retinopathy
HbA1c: Glycated haemoglobin
IVI: Intravitreal injection
logMAR: Logarithm of the minimal angle or resolution
MPL: Micropulse laser
REML: Random effects models
RPE: Retinal pigment epithelium
SML: Subthreshold micropulse laser
VF(D): Visual field (defect)
